# Supplementary material for: Physical activity in women attending a dissonance-based intervention after Roux-en-Y Gastric Bypass: A 2-year follow-up of a randomized controlled trial
Source: PLoS One. 2021 Nov 4;16(11):e0255556. doi: 10.1371/journal.pone.0255556 (PMC8568166; doi:10.1371/journal.pone.0255556)
Supplement: S1 Appendix — MVPA = moderate-to-vigorous physical activity; LPA = light physical activity. Presented as mean scores (standard errors) or numbers (percentage) for each variable, p-value for the difference between the groups at the two-year follow-up. Effect sizes at two years measured with Cohen’s d (95% CI). There are fewer participants with valid measurements at pre-RYGB than at the follow-ups, because not all participants had enough time to wear the accelerometer before their surgery. *PA-recommendations: ≥150 minutes of MVPA per week in non-bouts and 10-minute bouts. (DOCX) [file pone.0255556.s002.docx]

**S1 Appendix.** **Per protocol analysis of the physical activity intensities two years post-Roux-en-Y Gastric Bypass (RYGB) surgery for the women in the intervention group who received the intervention (attended ≥3 of 4 sessions) versus control group (standard care).**

| Accelerometer outcomes | Pre-RYGB,  Received intervention n=39 | Pre-RYGB,  Control group  n=46 | *p*-value | 2y post-RYGB,  Received intervention n=50 | 2y post-RYGB,  Control group  n=68 | *p*-value | Cohen’s *d*  (95% CI) |
| --- | --- | --- | --- | --- | --- | --- | --- |
| Mean wear time, hours/d (SE) | 14.5 (0.2) | 14.0 (0.2) | 0.109 | 15.4 (0.3) | 14.7 (0.2) | .091 | -.45 (-.82 to -.08) |
| Mean counts, min/d (SE) | 523.8 (31.0) | 564.1 (28.1) | 0.336 | 534.9 (23.8) | 579.9 (22.9) | .260 | .25 (-.12 to .62) |
| MVPA, min/d (SE) | 25.2 (2.9) | 24.5 (3.1) | 0.738 | 28.2 (2.5) | 27.1 (2.5) | .467 | -.06 (-.42 to .31) |
| LPA, min/d (SE) | 341.8 (15.5) | 359.3 (11.1) | 0.220 | 373.6 (11.7) | 394.0 (11.2) | .219 | .23 (-.14 to .60) |
| Sedentary time, min/d (SE) | 500.4 (17.8) | 455.5 (12.2) | 0.039 | 522.5 (17.2) | 458.8 (12.3) | .002 | -.58 (-.95 to -.20) |
| Mean steps, counts/d (SE) | 6112.0 (423.8) | 5971.5 (397.9) | 0.685 | 7514.6 (366.6) | 7387.8 (366.5) | .503 | -.04 (-.41 to .32) |
| Meeting PA-recommendations*, n (%) | 21 (53.9) | 20 (43.5) | 0.340 | 32 (64.0) | 37 (54.4) | .296 | -.19 (-.56 to .17) |
| Meeting PA-recommendations in ≤10-min bouts*, n (%) | 6 (7.1) | 4 (8.7) | 0.522 | 10 (20.0) | 13 (19.1) | .905 | -.02 (-.39 to .34) |

MVPA = moderate-to-vigorous physical activity; LPA = light physical activity. Presented as mean scores (standard errors) or frequency (percentage) for each variable, *p*-value (Kruskal-Wallis H test) for the difference in medians between the groups at the two-year follow-up. Effect sizes at two years measured with Cohen’s d (95 % CI). There are fewer participants with valid measurements at pre-RYGB than at the follow-ups, because not all participants had enough time to wear the accelerometer before their surgery. *PA-recommendations: ≥150 minutes of MVPA per week in non-bouts and 10-minute bouts.
