# Supplementary material for: Physical activity in women attending a dissonance-based intervention after Roux-en-Y Gastric Bypass: A 2-year follow-up of a randomized controlled trial
Source: PLoS One. 2021 Nov 4;16(11):e0255556. doi: 10.1371/journal.pone.0255556 (PMC8568166; doi:10.1371/journal.pone.0255556)
Supplement: S2 Appendix — MVPA = moderate-to-vigorous physical activity; LPA = light physical activity. Presented as mean scores (standard errors) or numbers (percent) for each subscale, p-value for the difference between the two groups at pre-RYGB and two-years post-RYGB surgery. Effect sizes at 2 years measured with Cohen’s d (95% CI). There are fewer participants with valid measurements at pre-RYGB than at the follow-ups, because not all participants had enough time to wear the accelerometer before their surgery. *PA-recommendations: ≥150 minutes of MVPA per week in non-bouts and 10-minute bouts. (DOCX) [file pone.0255556.s003.docx]

**S2 Appendix. Sensitivity analysis for baseline (pre-surgery) and two-years follow-up measures of the physical activity intensities (measured by the GT3X+ accelerometers) among the women, undergoing Roux-en-Y Gastric Bypass (RYGB) surgery, in the intervention group and control group who had ≥5 valid accelerometer measurement days.**

| Accelerometer outcomes | Pre-RYGB,  Intervention  (n=67) | Pre- RYGB,  Control  (n=41) | *p*- value | 2y post- RYGB, Intervention  (n=92) | 2y post- RYGB,  Control  (n=64) | *p*- value | Cohen’s *d*  (95% CI) |
| --- | --- | --- | --- | --- | --- | --- | --- |
| Mean wear time, hours/d (SE) | 14.4 (0.1) | 14.1 (0.1) | .239 | 15.3 (0.2) | 14.7 (0.2) | .293 | .33 (.01 to .65) |
| Mean counts, min/d (SE) | 556.0 (23.7) | 572.8 (28.6) | .646 | 562.5 (18.0) | 582.6 (24.2) | .609 | -.11 (-.43 to .21) |
| MVPA, min/d (SE) | 26.7 (2.2) | 25.9 (3.3) | .584 | 29.0 (1.8) | 27.5 (2.6) | .359 | .08 (-.24 to .40) |
| LPA, min/d (SE) | 356.3 (10.6) | 365.2 (10.3) | .571 | 388.2 (9.4) | 395.8 (11.6) | .351 | -.08 (-.40 to .24) |
| Sedentary time, min/d (SE) | 482.4 (12.1) | 457.8 (12.0) | .196 | 500.6 (12.5) | 460.8 (12.8) | .047 | .35 (.03 to .67) |
| Mean steps, counts/d (SE) | 6159.5 (288.3) | 6244.1 (408.2) | .897 | 7663.8 (275.8) | 7489.0 (383.8) | .451 | .06 (-.26 to .38) |
| Meeting PA-recommendations*, n (%) | 38 (56.7) | 19 (46.3) | .295 | 62 (67.4) | 35 (54.7) | .110 | .26 (-.06 to .58) |
| Meeting PA-recommendations in ≤10-min bouts*, n (%) | 6 (9.0) | 4 (9.8) | .889 | 16 (17.4) | 13 (20.3) | .645 | -.07 (-.39 to .24) |

MVPA = moderate-to-vigorous physical activity; LPA = light physical activity. Presented as mean scores (standard errors) or frequency (percent) for each subscale, *p*-value (Kruskal-Wallis H test) for the difference in medians between the two groups at baseline and two-years post-RYGB surgery. Effect sizes at 2 years measured with Cohen’s d (95 % CI). There are fewer participants with valid measurements at pre-RYGB than at the follow-ups, because not all participants had enough time to wear the accelerometer before their surgery. *PA-recommendations: ≥150 minutes of MVPA per week in non-bouts and 10-minute bouts.
