# Supplementary material for: Physical activity in women attending a dissonance-based intervention after Roux-en-Y Gastric Bypass: A 2-year follow-up of a randomized controlled trial
Source: PLoS One. 2021 Nov 4;16(11):e0255556. doi: 10.1371/journal.pone.0255556 (PMC8568166; doi:10.1371/journal.pone.0255556)
Supplement: S1 File — (DOC) [file pone.0255556.s004.doc]

Bilaga 2

Research plan

**Effects on eating behavior, body esteem, social adjustment and quality of life of a randomized, pre-operative, dissonance-based intervention for female gastric bypass patients.**

Finn Rasmussen^1^, Ata Ghaderi^2^, Erik Näslund^3^, Anders Thorell^4^ and Mikaela Willmer^1^

^1^Departments of Public Health Sciences, ^2^Department of Clinical Neurosciences and ^3^Department of Clinical Sciences, Danderyd Hospital, Karolinska Institutet, and ^4^Department of surgery, Ersta Hospital, Stockholm

**Background**

The global prevalence of obesity has more than doubled since 1980, with 200 million men and nearly 300 million women classified as obese in 2008 . In Sweden, approximately 50% of men and 40% of women are classified as overweight or obese . Obesity is associated with a number of co-morbidities such as type 2-diabetes, cardiovascular disease and some cancers . However, traditional weight loss methods, such as dietary restriction and increased physical activity, have been shown to have only a modest long-term effect on weight . Bariatric surgery, on the other hand, has been shown to result in long-term weight loss in the majority of patients, decreased all-cause mortality, improvement of many traditional cardiovascular risk factors and, in many cases, remission of type 2-diabetes, as well as improved health-related quality of life among a majority of patients .

The number of bariatric procedures carried out in Sweden has increased from 790 in 1997 to 7900 in 2012 . Gastric bypass (GBP) is the most common surgery method used in Sweden to treat severe obesity. It involves both restrictive and malabsorptive techniques. A small pouch is created in the proximal part of the stomach, and is then connected directly to the middle portion of the jejunum, bypassing the rest of the stomach and the duodenum. GBP necessitates major and permanent changes in the individual’s eating behaviours and food choices, such as eating very small, frequent meals, eating very slowly, and avoiding foods high in sugar and fat. The dietary changes required after GBP, coupled with the patient’s large weight loss, will have an impact on her eating habits and most likely also on her body image.

With the vast majority of GBP procedures in Sweden now being performed laparoscopically, with low rates of complications and post-operative mortality , our attention is starting to turn to the non-surgical aspects of GBP, which are not as well explored and where there may be room for improvement.

Although the majority of GBP patients experience major and long-term weight loss and improved mental and physical health, a sizeable minority of patients either fail to lose a satisfactory amount of weight, or start regaining large amounts of weight early on after surgery. In most cases, this is not caused by surgical issues , but rather by difficulties with the psychosocial aspects of the life changes brought about by the procedure . These aspects could include changes in self-esteem, coping skills, and functioning of close relationships with one’s children and/or partner .

Previous research has shown that although the patient’s health-related quality of life improves after GBP, this is closely associated with the weight loss. The improvement in health-related quality of life typically peaks during the first year after surgery, when weight loss is most rapid, and then declines again as gradual weight regain starts to occur at 1-6 years post-surgery .

When GBP patients fail to lose the expected amount of weight, or start to regain relatively early on after surgery, it is often due to the patient’s inability to adhere to the prescribed dietary recommendations . A relatively common behavioural problem after surgery is “grazing” – described by Saunders as “repeated episodes of consumption of smaller quantities of food over a long period of time with accompanying feelings of loss of control” . It seems to be especially common in patients who experience binge eating episodes before surgery, but may also affect patients who do not report bingeing pre-surgery.

When it comes to body esteem, it tends to improve after GBP, although it, like health-related quality of life, seems to follow the trajectory of weight loss . It is easy to imagine a negative spiral experienced by GBP patients as their weight loss slows down or weight regain occurs – shame and stigmatisation leading to increased sedentary and passive behaviours and avoidance of physical activity, which in its turn leads to even greater problems with body esteem and weight regain. It is therefore important to create efficient preventive measures to avoid this scenario.

It seems rational to provide patients with the tools for changing dysfunctional eating behaviours and issues with body esteem before surgery, rather than waiting until afterwards. However, there is very little research exploring how this may be achieved. Kalarchian et al conducted a preoperative lifestyle intervention which partly focused on addressing dysfunctional eating behaviours . However, the aim of that intervention was to achieve preoperative weight loss through dietary restriction and increased physical activity. Leahey et al randomized patients with problematic eating behaviours to receive a 10-week intervention, aimed at reducing these behaviours, either before or after surgery . However, only 3 of the 21 patients randomized to receive the intervention pre-operatively completed it, and no results are reported in terms of reduction in undesirable eating behaviours.

Thus, the overreaching goal of the current study is to find ways of optimising the outcome for the GBP patient, not only in terms of weight loss but also in terms of eating behaviour, body esteem, quality of life and social adjustment. In the current study we intend to investigate the effects of a four-session, pre-surgery, dissonance-based intervention on female GBP patients’ social adjustment and quality of life.

**Specific research questions**

- What are the effects on eating behavior and body esteem of a randomized, pre-operative, dissonance-based intervention for female gastric bypass patients?
- What are the effects on social adjustment and quality of life of a randomized, pre-operative, dissonance-based intervention for female gastric bypass patients?

**Methods**

We will address the research questions within the framework of a randomized controlled trial (RCT) of the effects of a pre-surgery, dissonance-based intervention. Women eligible for participation in the study will be identified from the waiting list for GBP surgery at two major surgical clinics in Sweden. The inclusion criteria are being severely obese (BMI ≥ 35kg/m^2^), being able to understand and speak Swedish and the absence of any serious chronic disease, e.g. stroke of or myocardial infarction.

The proposed RCT will assess the longitudinal changes in the women’s eating behaviour, body esteem, social adjustment and quality of life. It is anticipated that the women initially will show improved body image and self-esteem. We will of course also investigate any changes in the women’s BMI and fat mass.

At all clinical follow-up visits the women taking part in this study will be weighed, and their height and waist circumference measured. Bio-electric impedance (BIA) will be used to assess fat mass and fat-free mass. They will also be given questionnaires to complete at clinical visits. The women will be asked to complete the Three-Factor Eating Questionnaire (which measures restrained eating, emotional eating and uncontrolled eating) , the Hospital Anxiety and Depression Scale and the Karolinska Sleep Questionnaire.

The follow-up visits will capture any short-term changes. It is common for the GBP patient to experience a kind of “honey moon period” during the first 6-18 months after surgery, when weight loss is rapid. Any problems – physical or mental – are more likely to emerge after this period, when there might be weight regain, or a sense that the mind has not kept up with the change in appearance. However, if the women improve with respect to wellbeing and quality of life, it would be an added positive effect of GBP and a contribution to the current health economic evaluations of the procedure’s cost efficiency.

A total of 170 patients will be recruited from waiting lists for GBP at two major surgical clinics in Stockholm and randomised either to the intervention or to usual care. We expect 20% drop-out of patients during two years’ follow-up and power calculations show that 85 participants are needed in each group to attain a power of at least 0.80, with an expected moderate effect size (Cohen's d=50), and alpha set at .05. All patients will be asked to give informed written consent to participate in the study, and to give us access to their medical records. They will also complete the shortened version of the Social Adjustment Scale (which measures a range of aspects of social adjustment such as participation in the labour market and functioning in intimate relations) and the SF-36 , a widely-used instrument which measures health-related quality of life in several dimensions.

The intervention will start approximately two months before surgery, and will consist of four hour-long group sessions, during which a modified version of Stice’s dissonance-based treatment model for preventing eating disorders will be used. Dissonance-based interventions are based on the theory that psychological discomfort is created when a person attempts to hold two inconsistent cognitions at the same time. Cognitions are thus altered to create consistency, and this causes the person to change her behaviour. For example, in eating disorder prevention, if a participant in a group is asked to verbalize attitudes that oppose the thin body shape ideal, she is then likely to change her own private views to align with the stated ones. The model focuses on the participants actively engaging, rather than passively receiving information. As an example of an exercise that may be used in the current intervention, participants may be given a “letter” (written by the researchers) from a GBP patient who has had the surgery and is losing weight, but is still experiencing feelings of shame and stigma because of her size. This stops her from engaging in different social and physical activities. The participants are asked to give the woman advice, the idea being that they will express attitudes and strategies, which they are then more likely to adopt if they find themselves in a similar situation after their own GBP surgery. Apart from eating disorders, the model has been used for smoking cessation, substance abuse and obesity treatment with good results . In the current project, a psychologist will adapt it to GBP patients and test it under the supervision of Professor Ata Ghaderi (before the new PhD student has started).

The intervention is relatively short, inexpensive, and does not place any significant burden on the participants. It has shown positive results when used in other settings , and if good results can be reproduced for GBP patients, so that they obtain maximum benefit from their surgery, both in terms of weight loss and in terms of psychosocial wellbeing and social functioning, it would be of great economic and personal benefit both to the individual patients, and to society as a whole.

The control group will receive the usual follow-up care at the clinic where they had surgery, and like the intervention group, will be asked to complete the Social Adjustment Scale and the SF-36 before surgery and 6, 12, 18 and 24 months after surgery. In addition to the psychometric instruments already described, the intervention and control patients will complete the Body Esteem Scale and the Three-Factor Eating Questionnaire at all data collection points before and after GBP surgery as described above, and a questionnaire which specifically measures disordered eating after bariatric surgery at all follow-up data collections. Patients in the intervention and control groups will also have their weight, fat mass and waist circumference measured at similar time points before and after surgery and will be asked to report complications at all scheduled visits.

Analyses will focus on differences between intervention and control groups with respect to longitudinal changes from pre-surgery assessments to end of follow-up two years after GBP surgery in body esteem and eating behaviours, and whether the intervention helps those with existing disordered eating behaviours pre-surgery.

**References**

1. WHO, *Fact sheet 311. Obesity and overweight.*, 2012, WHO.

2. Neovius, M., A. Janson, and S. Rossner, *Prevalence of obesity in Sweden.* Obes Rev, 2006. **7**(1): p. 1-3.

3. Guh, D.P., et al., *The incidence of co-morbidities related to obesity and overweight: a systematic review and meta-analysis.* BMC Public Health, 2009. **9**: p. 88.

4. Brownell, K.D., *The humbling experience of treating obesity: Should we persist or desist?* Behav Res Ther, 2010. **48**(8): p. 717-9.

5. Anderson, J.W., et al., *Long-term weight-loss maintenance: a meta-analysis of US studies.* Am J Clin Nutr, 2001. **74**(5): p. 579-84.

6. Franz, M.J., et al., *Weight-loss outcomes: a systematic review and meta-analysis of weight-loss clinical trials with a minimum 1-year follow-up.* J Am Diet Assoc, 2007. **107**(10): p. 1755-67.

7. Edholm, D., et al., *Long-term results 11 years after primary gastric bypass in 384 patients.* Surg Obes Relat Dis, 2012.

8. White, S., et al., *Long-term outcomes after gastric bypass.* Obes Surg, 2005. **15**(2): p. 155-63.

9. *Scandinavian Obesity Registry Annual Report 2012*, 2013.

10. Sarwer, D.B., R.J. Dilks, and L. West-Smith, *Dietary intake and eating behavior after bariatric surgery: threats to weight loss maintenance and strategies for success.* Surg Obes Relat Dis, 2011. **7**(5): p. 644-51.

11. van Hout, G.C., S.K. Verschure, and G.L. van Heck, *Psychosocial predictors of success following bariatric surgery.* Obes Surg, 2005. **15**(4): p. 552-60.

12. Karlsson, J., et al., *Ten-year trends in health-related quality of life after surgical and conventional treatment for severe obesity: the SOS intervention study.* Int J Obes (Lond), 2007. **31**(8): p. 1248-61.

13. Saunders, R., *"Grazing": a high-risk behavior.* Obes Surg, 2004. **14**(1): p. 98-102.

14. Sarwer, D.B., et al., *Changes in quality of life and body image after gastric bypass surgery.* Surgery for Obesity & Related Diseases, 2010. **6**(6): p. 608-14.

15. Kalarchian, M.A., et al., *Preoperative lifestyle intervention in bariatric surgery: initial results from a randomized, controlled trial.* Obesity (Silver Spring), 2013. **21**(2): p. 254-60.

16. Leahey, T.M., et al., *When is the best time to deliver behavioral intervention to bariatric surgery patients: before or after surgery?* Surg Obes Relat Dis, 2009. **5**(1): p. 99-102.

17. Karlsson, J., et al., *Psychometric properties and factor structure of the Three-Factor Eating Questionnaire (TFEQ) in obese men and women. Results from the Swedish Obese Subjects (SOS) study.* International journal of obesity and related metabolic disorders : journal of the International Association for the Study of Obesity, 2000. **24**(12): p. 1715-25.

18. Lisspers, J., A. Nygren, and E. Soderman, *Hospital Anxiety and Depression Scale (HAD): some psychometric data for a Swedish sample.* Acta Psychiatr Scand, 1997. **96**(4): p. 281-6.

19. Gameroff, M.J., P. Wickramaratne, and M.M. Weissman, *Testing the Short and Screener versions of the Social Adjustment Scale-Self-report (SAS-SR).* Int J Methods Psychiatr Res, 2012. **21**(1): p. 52-65.

20. Sullivan, M., J. Karlsson, and J.E. Ware, Jr., *The Swedish SF-36 Health Survey--I. Evaluation of data quality, scaling assumptions, reliability and construct validity across general populations in Sweden.* Soc Sci Med, 1995. **41**(10): p. 1349-58.

21. Stice, E., et al., *Dissonance-based Interventions for the prevention of eating disorders: using persuasion principles to promote health.* Prev Sci, 2008. **9**(2): p. 114-28.

22. Freijy, T. and E.J. Kothe, *Dissonance-based interventions for health behaviour change: a systematic review.* Br J Health Psychol, 2013. **18**(2): p. 310-37.

23. Mendelson, B.K., M.J. Mendelson, and D.R. White, *Body-esteem scale for adolescents and adults.* J Pers Assess, 2001. **76**(1): p. 90-106.

24. Weineland, S., Alfonsson, S, Dahl, J, Ghaderi, A, *Development and validation of a new questionnaire measuring eating disordered behaviours post bariatric surgery.* Clinical Obesity, 2013. **2**: p. 160-167.
