# Supplementary material for: Physical activity in women attending a dissonance-based intervention after Roux-en-Y Gastric Bypass: A 2-year follow-up of a randomized controlled trial
Source: PLoS One. 2021 Nov 4;16(11):e0255556. doi: 10.1371/journal.pone.0255556 (PMC8568166; doi:10.1371/journal.pone.0255556)
Supplement: Attachment — Submitted filename: PONE-D-20-01663.docx [file pone.0255556.s005.docx]

**Physical activity in women attending a dissonance-based intervention after Roux-en-Y**

**Gastric Bypass: A 2-year follow-up of a randomized controlled trial**

Plos One 2020

**General comments**

In this paper, authors assessed the effect of a dissonance-based intervention conducted in the first months after RYGB on habitual physical activity up to two years after surgery. They conclude that no difference was observed between physical activity at the two-year follow-up between intervention and control groups. A previous paper by the same group described the effect of the intervention at the 1-year follow-up. Strengths of the study were to use a randomized design and to assess physical activity with accelerometers. The study is well written, and the references are up to date.

My only concern is about the statistical methods used. Authors compared physical activity outcomes between the two groups at each time point (pre-surgery, one-year and two-year post-surgery) instead of comparing longitudinal changes in physical activity. By doing so, they included only participants with valid data at the 2-year follow-up and therefore excluded many participants who had valid data at baseline but not at 2 years. Mixed models might be more appropriate when several time points are available.

**Specific comments**

**Abstract**

- Line 28: Is “baseline” a pre-surgery measure? If so, “before surgery” might be more precise than “at baseline”

**Introduction**

- Line 58-67: the references are up to date but the message of this paragraph is not clear to me. The expression “In addition” (line 65) does not seem appropriate to me, “In contrast” might be more appropriate here.

What I understand from this paragraph is that: 1) exercise training has been shown effective after bariatric surgery, 2) mixed results are found regarding the spontaneous change in physical activity after bariatric surgery, 3) exercise training leads to a small and transient increase in habitual physical activity, 3) physical activity counselling might be effective to increase physical activity.

I would suggest to make a clearer distinction between changes in physical activity related to bariatric surgery and those related to exercise training after bariatric surgery.

- Line 91: “objectively-measured” rather than “objective measured”

**Methods**

- Line 98-99: “we analysed how the intervention affected physical activity”

**Results**

- Tables do not fit in the pdf. I cannot comment the tables.
- The authors compared physical activity outcomes between intervention and control group at all times (Table 2). This method results in the exclusion of many participants who do no hate valid measures at the 2-year follow-up. It would have been more appropriate to compare changes in physical activity between groups. Mixed models are usually used in this situation.

**Discussion**

- Lines 307-309: Are the results similar in other contexts (eg in the studies cited in the introduction)?
