# Supplementary material for: Physical activity in women attending a dissonance-based intervention after Roux-en-Y Gastric Bypass: A 2-year follow-up of a randomized controlled trial
Source: PLoS One. 2021 Nov 4;16(11):e0255556. doi: 10.1371/journal.pone.0255556 (PMC8568166; doi:10.1371/journal.pone.0255556)
Supplement: Attachment — Submitted filename: Review.docx [file pone.0255556.s006.docx]

Thank you for the opportunity to review the manuscript entitled ‘Physical activity in women attending a dissonance-based intervention after Roux-en-Y- Gastric Bypass: A 2-year follow-up of a randomized controlled trial’. This is an interesting paper looking at objectively measured physical activity in a sample of women whom have undergone RYGB, this is a large sample given the population and topic area (physical activity) that the authors are researching in. It is also an area of research which is in its infancy and a useful addition to the literature.

General comments

Overall a good well written manuscript, I have the following questions I would like you to consider;

1. You have undertaken a dissonance-based intervention comprising four sessions that focus on different topic areas. One of these focus on physical activity, nowhere in this manuscript can I see how many of the individuals analysed attended this physical activity session. Surely it is important to know how many attended the physical activity session; you wouldn’t typically expect physical activity to increase when compared to normal care if they have not been given an additional physical activity intervention. Did you look at data from those who attended the physical activity session?
2. All tables were cut off so I was unable to read half of the table.

Specific comments

Page 1 line 58 – instead of ‘inconclusive’ should this be ‘limited’

Page 2 line 74 – remove for example

Page 2 line 77 – instead of ‘Also’ should this be ‘However’

Page 2 line 81 – change ‘surgeries’ to ‘surgery procedures’

Page 2 lines 87-90 – instead of ‘with the aim to’ potentially change to ‘aiming to’, this is a long sentence.

Page 5 line 98-99 – this sentence needs to be slightly reworded or add how after ‘analyse’

Page 5 line 109 – Was wearing the accelerometer optional? ‘ were offered to wear’ makes it sound optional

Page 6 line 117 Re-wording suggestion ‘ participants who had provided informed consent and baseline data…’

Page 6 line 127 Trained facilitators – include what the training comprised of and in what ‘teaching’ style was the programme delivered?

Page 6 line 135-136 – the participant was considered as having received the intervention if they attended three sessions was it any three sessions? If so state this.

Page 6 line 139-140 – I think it would be useful to mention the topics covered in the physical activity session.

Page 6 line 139-140 - Did you refine the programme based on the pilot feedback – this may also be worth mentioning

Page 7 line 144 – was the accelerometer worn on the dominant or non-dominant wrist? This should be reported.

Page 7 line 146 – why the 10 hour threshold? Is there a reference for this?

Page 9 table half missing and the variables in the table are not clear if they are baseline or 2 years post.

Page 10 line 225 – what do you mean by non bouts? 1 minute bouts?

Page 10-11 Table 2 format

Page 11 line 232 – the sentence starts with presented – what is this sentence referring to?

Page 11 line 244 – a sedentary time difference between groups of 64 minutes per day seems large, you have stated that it is not relevant from a clinical perspective… if this is correct you need to reference this. This is implying that the individuals are more sedentary as a result of attending the programme, is this correct?

Page 13 discussion – I think the difference in sedentary time (increase in intervention group) should be mentioned in the results and justified.

Page 13 line 286 – when referring to the other research, it would be useful to know when these studies were undertaken post-surgery was it 2 years or 6 months etc?

Page 13 line 294 – physical activity increased by how much?

Page 14 line 314 – attendance at at least one session, was this one session the physical activity session or not? If not why not? If not how many of those included attended that session?

If they did not attend the physical activity session this is likely why you didn’t see a change and previous research in this population reports exercise education alone is insufficient for increasing physical activity.

Limitation – may not have attended the physical activity session

You have mentioned you were likely not powered, have you looked at your data to see how many individuals you would have needed to be powered?

Fig 1 – include amount of individuals who attended the physical activity session
